# Overexpression of Semaphorin 3A is a Marker Associated with Poor Prognosis in Patients with Nasopharyngeal Carcinoma

**DOI:** 10.3390/microorganisms8030423

**Published:** 2020-03-17

**Authors:** Tomoko Imoto, Satoru Kondo, Naohiro Wakisaka, Pham Tahnh Hai, Noriko Seishima, Makoto Kano, Takayoshi Ueno, Harue Mizokami, Yosuke Nakanishi, Miyako Hatano, Kazuhira Endo, Hisashi Sugimoto, Makiko Moriyama-Kita, Tomokazu Yoshizaki

**Affiliations:** Division of Otolaryngology and Head and Neck Surgery, Graduate School of Medical Science, Kanazawa University, 13-1 Takara, Kanazawa, Ishikawa 920-8641, Japan; imoto@med.kanazawa-u.ac.jp (T.I.); wakisaka@med.kanazawa-u.ac.jp (N.W.); thanhhai.hpmu@gmail.com (P.T.H.); nseishima@med.kanazawa-u.ac.jp (N.S.); makoto-kano@med.kanazawa-u.ac.jp (M.K.); uenotaka@med.kanazawa-u.ac.jp (T.U.); haruesun@med.kanazawa-u.ac.jp (H.M.); nakanish@med.kanazawa-u.ac.jp (Y.N.); mhatano@med.kanazawa-u.ac.jp (M.H.); endok@med.kanazawa-u.ac.jp (K.E.); sugimohi@med.kanazawa-u.ac.jp (H.S.); tomoy@med.kanazawa-u.ac.jp (T.Y.)

**Keywords:** nasopharyngeal carcinoma, semaphorin, Epstein–Barr virus, latent membrane protein 1 (LMP1), prognosis

## Abstract

Semaphorins were discovered as guidance signals that mediate neural development. Recent studies suggest that semaphorin 3A (Sema3A), a member of the semaphorin family, is involved in the development of several cancers. This study aimed to analyze the association of Sema3A with the clinical features of nasopharyngeal carcinoma (NPC), an Epstein–Barr virus-associated carcinoma, and the Epstein–Barr virus primary oncogene latent membrane protein 1 (LMP1). The expression of Sema3A and LMP1 was immunohistochemically examined in the 35 NPC specimens. The mean expression scores for Sema3A and LMP1 were 20.8% ± 14.5% and 13.9% ± 14.8%, respectively. The expression of Sema3A significantly correlated with that of LMP1 (r = 0.41, *p* = 0.014). In addition, the Sema3A high cohort showed significantly poorer prognosis than the Sema3A low cohort. Sema3A expression was higher in the LMP1-positive KH-1 and KR-4 cell lines compared to the LMP1-negative HeLa cells. Overexpression of LMP1 in the LMP1-negative AdAH cell line upregulated Sema3A expression, both at the transcriptional and translational level. Finally, Sema3A expression was associated with poor prognosis in patients with NPC. Our data suggest that LMP1 induces the expression of Sema3A, which may promote tumor progression in NPC.

## 1. Introduction

Nasopharyngeal carcinoma (NPC) typically has invasive and metastatic features clinically. Despite recent advances in radiation techniques and chemotherapy, NPC patients who develop distant metastasis still have a poor survival rate [1]. Epstein–Barr virus (EBV) is the causative agent in most cases of NPC [2,3]. EBV is an oncogenic herpesvirus that encodes the latent membrane protein (LMP1) as its principal oncoprotein. LMP1 is expressed in NPC tumor tissues and has been shown to induce transformation, inhibit differentiation, and promote the migration of epithelial cells [4,5]. In addition, we have shown that LMP1 induces a variety of invasive and angiogenic factors using NPC cell lines [6,7,8]. Semaphorins were initially discovered in the embryonic nervous system [9] and found to play a role in neuronal network formation and neurite outgrowth [10,11]. Although semaphorins are involved in the development of the nervous system, they are also expressed in a wide range of non-neural cells and modulate numerous physiological and pathological processes [11,12,13]. Some members of the class3 semaphorins are upregulated in cancer [14,15,16], including Semaphorin3A (Sema3A), which is closely associated with cancer invasion and metastasis [17,18]. Sema3A alters cancer cell behavior directly by affecting cell migration and growth [17,18], and is frequently overexpressed in glioblastoma, pancreatic, lung, and prostate cancer [19,20,21,22]. Its increased expression positively correlates with the grade of malignancy, cell migration, metastatic potential, and poor prognosis [20,23]. Furthermore, Sema3A directly decreases focal adhesions, induces cancer cell invasion in vitro, and modulates tumor-associated macrophages [17,18]. Moreover, Sema3A can affect cell phenotype indirectly by interfering with tumor angiogenesis and the immune response [12,13]. On the other hand, several reports suggest that Sema3A may be a potent tumor suppressor in oral and lung cancers [23,24]. Whether Sema3A functions as a tumor suppressor or tumor promoter is still unclear and may be cancer specific. Since the role of Sema3A in the pathogenesis of NPC is unknown we investigated Sema3A expression in NPC using biopsy specimens. We also analyzed Sema3A expression and clinicopathological factors, especially clinical prognosis. Finally, we examined whether LMP1 signaling regulates Sema3A expression using nasopharyngeal cell lines.

## 2. Materials and Methods

### 2.1. Cell Culture

KH-1 is an EBV-positive type II cell line derived from the fusion of KR-4 (an EBV-positive type III lymphoblastoid cell line) and HeLa cells (human cervical cancer cell line) as previously described [25]. AdAH is an EBV-negative human nasopharyngeal cell line [25]. AdAH-pFB-LMP1 and AdAH-pFB-Neo have been described previously [6]. KR-4 cells were maintained in Roswell Park Memorial Institute 1640 medium containing 10% fetal bovine serum (FBS), penicillin, and streptomycin. The other cell lines were maintained in Dulbecco’s modified Eagle medium (DMEM) with 10% FBS, penicillin, and streptomycin. AdAH-pFB LMP1 and AdAH-pFB-Neo were maintained in DMEM with 10% FBS, penicillin, streptomycin, and 700 μg/mL G418 (Life Technologies, Grand Island, NY).

### 2.2. Antibodies and Transfection

Mouse monoclonal antibody against LMP1 was purchased from DAKO (Glostrup, Denmark). Rabbit monoclonal antibodies against Sema3A were purchased from Abcam (Cambridge, MA, USA), and mouse monoclonal antibody against α-tubulin was purchased from Sigma Aldrich (St. Louis, MO, USA). The pcDNA3-based LMP1 construct has been previously described [8]. For transient expression, cells were transfected for 48 h with 2 μg of the appropriate plasmids using the Effectene Transfection Reagent (Qiagen, Valencia, CA) following the manufacturer’s instructions.

### 2.3. RT-PCR Analysis

Total RNA was isolated using the RNeasy Mini Kit (QIAGEN, Hilden, Germany) and reverse transcribed using SuperScript III (ThermoFisher Scientific, Waltham, MA). The resulting cDNAs were used for the amplification of Sema3A and GAPDH cDNA using Taq DNA polymerase (Takara Bio, Ootu, Japan). The following primer sets were used for the amplification of Sema3A and Glyceraldehyde-3-phosphate dehydrogenase (GAPDH): Sema3A, 5′-GGCATATAATCAGACTCACTTGTACGC-3′ (forward) and 5′-CTTGCATATCTGACCTATTCTAGCGTG-3′ (reverse); GAPDH, 5′-TGGTCACCAGGGCTGCTTTTA-3′ (forward) and 5′-TCCTGGAAGATGGTGATGGGATTT-3′ (reverse). PCR reactions were performed using the following conditions: Sema3A: 94 °C for 10 min, 30 cycles at 94 °C for 30 sec, 50 °C for 30 sec, and 72 °C for 30 sec, and a final 5 min 72 °C extension. The PCR products were analyzed by 1.2% agarose gel electrophoresis with ethidium bromide and a UV light transilluminator.

### 2.4. Western Blotting

Whole-cell lysates were prepared in 100 μL of radioimmunoprecipitation assay (RIPA) buffer (50 mmol/L Tris-HCl (pH 7.5), 150 mmol/L NaCl, 1% NP40, 0.50% sodium deoxycholate, 0.10% sodium dodecyl sulfate (SDS), 0.2 mmol/L sodium orthovanadate, 0.1 mol/L NaF, and 5 μg fluoride/mL), denatured in SDS loading buffer and boiled for 5 min. Samples were separated by SDS-polyacrylamide gel electrophoresis (SDS-PAGE) and transferred to nitrocellulose membranes (GE Healthcare Japan, Tokyo, Japan). Membranes were blocked with 5% milk in Tris-buffered saline-Tween 20 (TBST) and incubated overnight at 4 °C with primary antibodies. Membranes were then washed and incubated with appropriate horseradish peroxidase-conjugated secondary antibodies for 1 h at room temperature. Membranes were washed again, and bands were visualized with enhanced chemiluminescence reagent (GE Healthcare Japan). Band intensity was quantified using the Image J software (http://rsb.info.nih.gov/nih-image/).

### 2.5. Patient Characteristics

We obtained 35 tumor specimens from patients with NPC who had been diagnosed at the Division of Otolaryngology at Kanazawa University Hospital and other branch hospitals between May 1999 and December 2007 (Table 1). The patients included 27 men and 8 women, aged 30–83 years (mean age, 58.7 years), and classified histologically as follows: 3 keratinizing squamous cell carcinoma (type I), 19 non-keratinizing carcinoma (type II), and 13 undifferentiated carcinoma (type III) based on the 8th TNM classification system of the International Union Against Cancer, 2016 [26]. There were 2 patients with stage I, 10 patients with stage II, 16 patients with stage III, and 7 patients with stage IV cancer. All specimens were positive for EBER-ISH (Epstein–Barr encoding region-in-situ-hybridization) [27].

Of the 35 patients, 25 received radiotherapy with concurrent cisplatin and 5-fluorouracil chemotherapy. Six patients received alternating cisplatin-based chemo-radiotherapy, as described previously [28], and 4 patients received only radiotherapy because of age (>70 years) or renal dysfunction. The accumulated radiation dose to the nasopharynx was 60–70 Gy in all cases. The median time of follow-up was 3.53 years (range 0.11–9.18 years). The survival period was calculated according to the date of the initial treatment. This study design was approved by the Ethical Committee of Kanazawa University, and informed consent was obtained from all patients before enrollment.

### 2.6. Immunohistochemical Analysis

Primary NPC paraffin-embedded specimens were used for the immunohistochemical analysis of Sema3A and LMP1 expression. From each block of tissue embedded in paraffin, 3 µm-thick sections were prepared. Deparaffinized sections were treated with 3% hydrogen peroxide for 10 min to inactivate endogenous peroxidase activity. The sections were incubated with a protein blocker (Dako, Glostrup, Denmark) for 20 min and incubated at 4 °C overnight with anti-Sema3A antibody or anti-LMP1 antibody. The sections were washed three times with phosphate-buffered saline (PBS, pH 7.2). After washing, the sections were exposed to an Envision^+^ secondary antibody (Dako) for 30 min. The reaction products were developed by immersing the sections in a 303-diamidobenzidine tetrahydrochloride solution and counterstained with hematoxylin.

For dual fluorescence immunostaining, 5 NPC specimens that were found to express LMP1 by single immunostaining were used. Dual staining for Sema3A and LMP1 was performed to assess the location of cancer cells expressing LMP1 in each specimen. Deparaffinized sections were microwaved in a citrate buffer (pH 6.0) for 20 min and then incubated in a protein blocker (Dako) for 20 min. The specimens were incubated overnight at 4 °C with primary antibodies. The reaction product was visualized with goat anti-mouse Alexa Fluor 594 and anti-rabbit Alexa Fluor 488 IgG secondary antibodies (1:500; Thermo Fisher Scientific). Specimens were counterstained with 4′,6-diamidino-2-phenylindole (DAPI; Thermo Fisher Scientific) and observed under a microscope.

### 2.7. Evaluation of Specimens

All slides were evaluated independently by two investigators (T.I. and S.K.) without previous knowledge of the clinical data and were then reviewed with the others. In each case, two arbitrary microscopic fields (100X) containing > 200 tumor cells were evaluated. After counting both immunoreactive cells and the total number of tumor cells, the average frequency of immunoreactive cells was calculated. The average percentage of immunoreactive cells was defined as the expression score and was used for statistical analysis.

### 2.8. Statistical Analysis

To test the association between Sema3A expression, LMP1 expression, and other clinicopathologic characteristics, one-way ANOVA test, unpaired *t*-test, and Mann–Whitney U test were performed. Overall survival according to Sema3A expression was displayed graphically as a Kaplan–Meier curve. The observed differences in survival were analyzed by the log-rank test. The *p* values < 0.05 were considered statistically significant. Univariate and multivariate analyses were performed using the Cox proportional hazard regression model for the variables (age, gender, T, N, and M classification, stage, histologic type, and initial therapy). Factors with *p* values ≤ 0.05 in the univariate models were included in the multivariate analysis. IBM SPSS Statistics version 23 (IBM, Armonk, NY, USA) was used for data analysis.

## 3. Results

### 3.1. LMP1 and Sema3A Expression in Cell Lines

**Figure 1:**
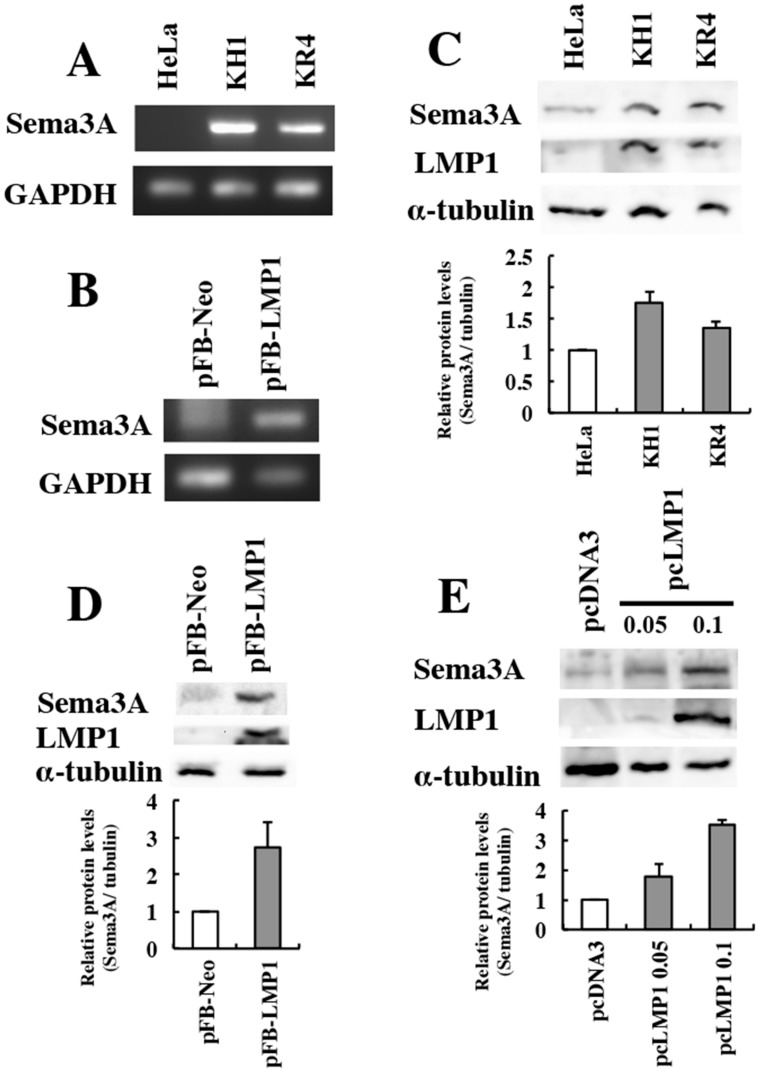
LMP1 upregulates Sema3A expression in human cell lines. KR-4 is an Epstein-Barr virus-positive lymphoblastoid cell line. The KH-1 cell line is derived by the fusion of KR-4 and HeLa cells. AdAH cells expressing pFB-Neo or pFB-LMP1 were also analyzed. (**A**,**B**) Sema3A mRNA expression was analyzed by RT-PCR. (**C**,**D**) Expression of Sema3A, LMP1, and tubulin was analyzed by Western blotting. The analysis was performed twice. The ratio of the intensity of Sema3A to that of α-tubulin, as measured by the Image J software, is shown as the relative expression level. (**E**) AdAH cells were transiently transfected with increasing amounts of pcLMP1 (0.05, 0.1 μg) for 48 h and analyzed by Western blotting. The analysis was performed twice. The ratio of the intensity of Sema3A to that of α-tubulin is shown as a relative Sema3A expression level.

To determine whether the expression of Sema3A is higher in LMP1-positive cell lines, we used KH-1, KR-4, and HeLa cells. KH-1 is an LMP1-positive epithelial cell line formed by the fusion of an EBV-positive lymphoblastoid cell line (KR-4) and EBV-negative epithelial cell line (HeLa) [29]. Sema3A mRNA expression was significantly higher in KR-4 and KH-1 cells, both of which express LMP1, than in LMP1-negative HeLa cells (Figure 1A). Based on this result, we next checked the Sema3A mRNA expression in cell lines stably expressing LMP1. We used AdAH-pFB-LMP1, a stable LMP1-expressing clone, and compared it with the control clone AdAH-pFB-Neo. As shown in Figure 1B, Sema3A mRNA expression was higher in the LMP1-positive clones. Sema3A mRNA expression appeared to correlate with LMP1 expression in the different cell lines (Figure 1A,B).

Since we found that LMP1-positive cells express Sema3A mRNA, we next determined the protein levels of Sema3A in these cells by Western blotting. The protein levels of Sema3A were about 1.5-fold higher in KH1 and KR4 which express LMP1 compared to HeLa cells (Figure 1C). Similarly, the levels of Sema3A protein were more than 2-fold higher in AdAH-pFB-LMP1 cells compared to AdAH-pFB-Neo control cells (Figure 1D). These results revealed that the levels of Sema3A protein appeared to correspond to the levels of LMP1 in the different cell lines.

Finally, to test whether transient expression of LMP1 can induce Sema3A expression, AdAH cells were transiently transfected with different amounts of an LMP1 expression plasmid (0.05 μg and 0.1 μg). LMP1 induced the expression of Sema3A protein depending on the amount of transfected LMP1 expression plasmid (Figure 1E: pcDNA3 vs. pcLMP1 0.05 μg: 1.75-fold; pcDNA3 vs. pcLMP1 0.1 μg: 3.4-fold). These results indicate that LMP1 induces the expression of Sema3A mRNA and protein in a dose-dependent manner.

### 3.2. LMP1 and Sema3A Expression in NPC Tissues

**Figure 2:**
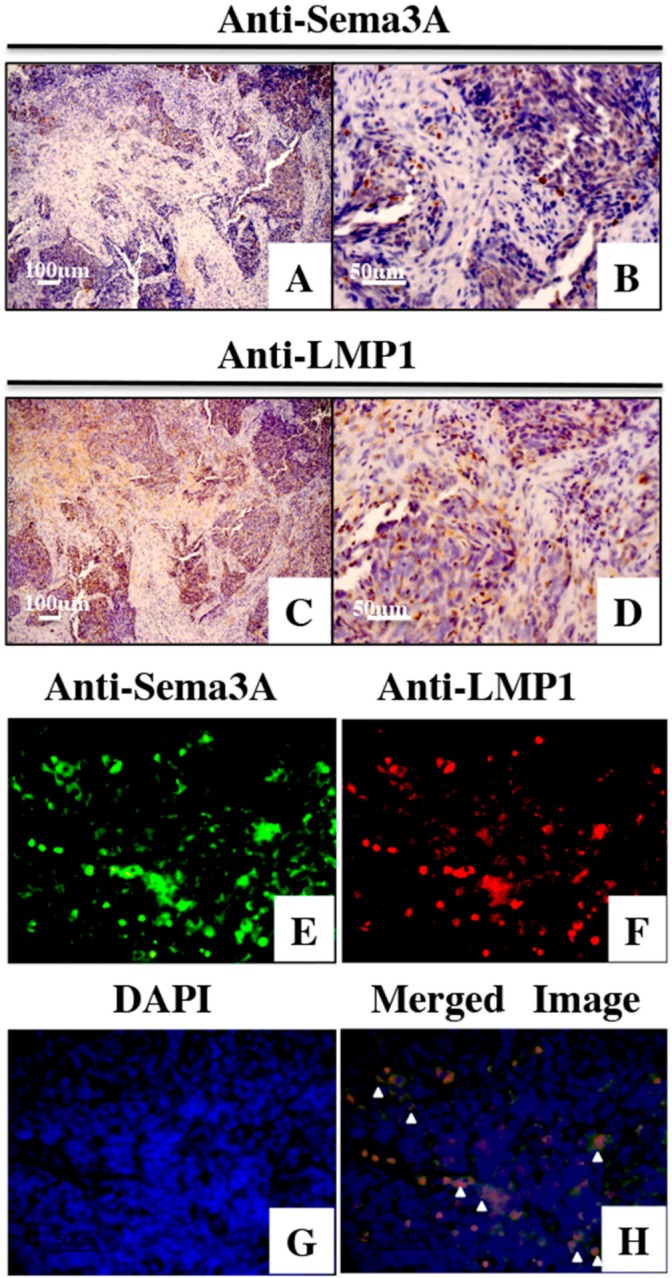
Immunostaining for Sema3A and LMP1 in nasopharyngeal carcinoma (NPC) tissues. (**A**,**B**) Immunostaining for Sema3A is shown. Dark brown staining indicates the expression of Sema3A. (**C**,**D**) Immunostaining for LMP1 is shown. Dark brown staining indicates the expression of LMP1. (**E**–**G**) Dual fluorescence immunostaining of Sema3A (**E**), LMP1 (F), and DAPI (**G**) in NPC tissues. (**H**) Merged image of Sema3A and LMP1. Arrowheads indicate the colocalization of LMP1 and Sema3A. Original magnification: 100× (**A**,**C**), 400× (**B**,**D**).

**Figure 3:**
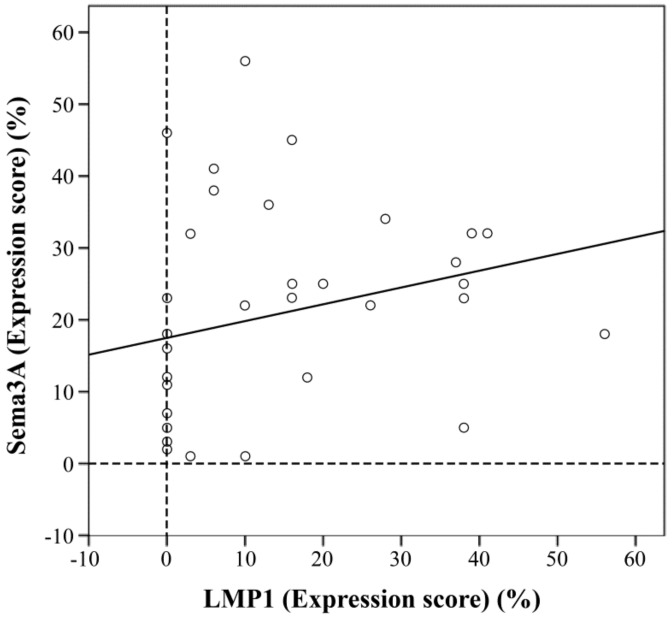
Correlation between Sema3A and LMP1 expression in NPC. The Sema3A and LMP1 expression scores for the 35 NPC cases are plotted. Sema3A expression significantly correlated with LMP1 expression according to the Spearman correlation coefficient (r = 0.42, *p* < 0.001).

To examine whether Sema3A is expressed in NPC tissues, we checked the protein levels of Sema3A and LMP1 by immunohistochemical staining (Figure 2). Sema3A was found to be localized to the cytoplasm and nucleus (Figure 2A,B). Moreover, Sema3A was also expressed in normal nasopharyngeal cells around the tumor cells (Figure 2B). LMP1 was primarily localized to the cytoplasm (Figure 2C,D). The mean expression scores for Sema3A and LMP1 were 20.8 ± 14.5% and 13.9 ± 14.8%, respectively. Of the 35 specimens, 18 (51.4%) were categorized as Sema3A positive and 22 cases (62.8%) were LMP1 positive. To evaluate the relationship between LMP1 and Sema3A expression, we analyzed the expression of LMP1 and Sema3A in the same tissue sample. Dual fluorescence immunostaining of LMP1 and Sema3A was performed (Figure 2E–H). Several cells that expressed LMP1 also expressed Sema3A in the NPC specimens (Figure 2H, arrow heads). Finally, we determined whether the expression scores of LMP1 and Sema3A were correlated. As shown in Figure 3, there was a moderate positive correlation between the expression of LMP1 and Sema3A (r = 0.42, *p* < 0.05). The levels of LMP1 and Sema3A in each sample are shown in Supplementary Table S1.

### 3.3. Relationship between Clinicopathological Features and the Expression of Sema3A

The association between the patients’ clinicopathological features and the expression score for LMP1 and Sema3A is shown in Table 1. TNM classification and stage grouping were divided into two categories, namely (stage I and II) or (stage III and IV) [26]. The lesional lymph node stages were classified as (N0) or (N1, N2, and N3). Distant metastasis at the initial visit were classified as M0 or M1. Clinical stages were divided into (stage I and II) and (stage III and IV). There was no correlation between the Sema3A or LMP1 expression scores and the clinicopathological features of the patients. However, the data featured in Table 1 reveal that men had significantly higher levels (*p* ˂ 0.02) of Sema3A as compared with women.

### 3.4. Relationship between Patient Prognosis and Expression of Sema3A and LMP1

**Figure 4:**
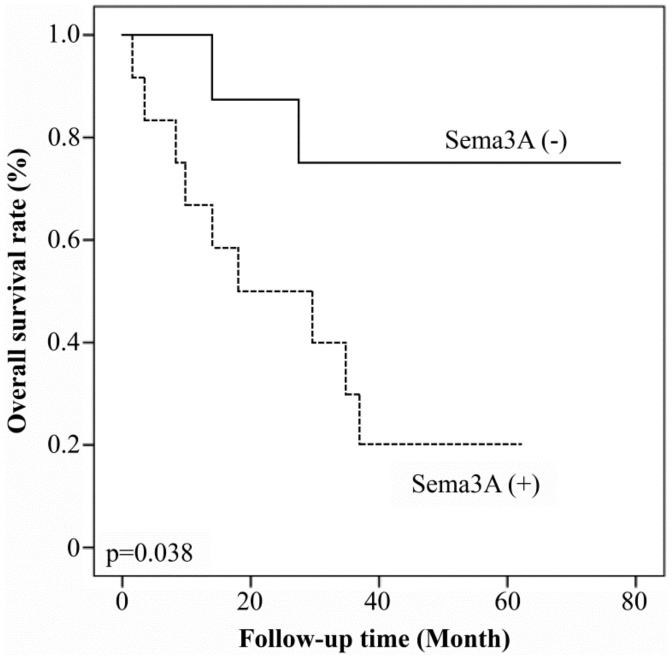
Sema3A expression and the survival rates of 35 patients with NPC. The relationship between Sema3A expression and patient prognosis was examined using Kaplan–Meier analysis.

We evaluated the prognostic value of Sema3A and LMP1 expression in patients with NPC. Patients with Sema3A-positive tumors showed worse overall survival rates than those with Sema3A-negative tumors (*p* = 0.038, Figure 4). Next, we evaluated whether Sema3A expression could represent an independent prognostic factor by Cox regression analysis. First, the association between the treatment protocol and patient prognosis was examined, and treatment differences were shown not to affect the prognosis of NPC patients, according to the results of a univariate analysis (*p* = 0.99, Table 2). In addition, a univariate Cox regression analysis did not show a significant association of age (≥50), gender, tumor status, lymph node status, stage, and LMP1-positivity. Univariate Cox regression analysis showed that distant metastasis and Sema3A expression represented significant hazards (Table 2). Finally, a multivariate Cox regression analysis revealed that the expression of Sema3A and distant metastasis were factors associated with poor prognosis (Table 2).

## 4. Discussion

This study is the first to show Sema3A expression in NPC and its association with poor prognosis. Some reports suggest that Sema3A acts as a tumor suppressor in certain cancers [30,31]. In contrast, other researchers have shown that Sema3A alters tumor cell behavior directly by influencing migration and growth, or indirectly by interfering with tumor angiogenesis and the immune response [1,24,32,33]. Our findings agree with the results derived from other solid tumors, including pancreatic, lung, and prostate cancers [20,21,23]. Furthermore, one study has shown a positive correlation between Sema3A and the metastatic potential of hepatocellular carcinoma [34]. Müller et al. have shown that high expression of Sema3A promotes tumor dissemination and metastasis, which correlates with worse prognosis in pancreatic cancer [20]. These studies support our results that Sema3A expression affects the prognosis of NPC. A recent study showed Sema3A expression in a variety of cancer cell lines and demonstrated that secretion of Sema3A by tumor cells enables manipulation of their microenvironment through suppression of T-cell activation [35]. This study also supports our results that Sema3A expression leads to tumor progression. When stratifying the expression levels of Sema3A by gender, males were shown to have elevated levels of Sema3A compared to females. As there are no reports confirming this finding, further studies with larger cohorts are required to better elucidate the gender bias in Sema3A expression.

Over the past decade we have reported on LMP1’s contribution to cancer invasion and metastasis [6,7,8]. We also examined LMP1 expression in NPC tissues and its association with Sema3A. We observed a moderate positive correlation between Sema3A expression and LMP1 expression by immunohistochemical analysis. However, there was a moderate positive correlation between Sema3A expression and LMP1 expression by immunohistochemical analysis. In contrast, we found that LMP1 strongly induces Sema3A expression in vitro using LMP1-positive cell lines. We speculate that this discrepancy may be caused by a variety of genetic/environmental stimuli other than LMP1 expression. For example, we have reported that the pro-angiogenic vascular endothelial growth factor (VEGF) is highly expressed in NPC tissues even though VEGF and LMP1 expression is not correlated [36]. Since VEGF stimulation upregulates Sema3A expression in certain cell lines [30], the positive transcriptional loop between VEGF and Sema3A might explain the discordance between the immunohistochemical and in vitro analysis of Sema3A and LMP1.

## 5. Conclusions

Results obtained from NPC tumor sections and from cell lines transiently expressing LMP1 suggest that LMP1 induces Sema3A expression, which in turn may promote tumor progression in NPC patients. Sema3A may therefore serve as a potential prognostic marker for NPC.

## Figures and Tables

**Table 1:** Relationship between the clinicopathological features of the patients and expression of LMP1 and Sema3A.

Total Patients	35	LMP1 Score		Sema3A Score	
		(mean SD)	*p* Value	(mean SD)	*p* Value
Gender					
Male	27	16.6 ± 14.8		30.2 ± 10.7	
Female	8	13.1 ± 15.9	0.59	18 ± 14.4	0.02
Age					
>50years	26	14.9 ± 15.9		64 ± 13.2	
<50years	9	11.1 ± 15.1	0.5	44.2 ± 17.2	0.51
T classification					
T1, 2	16	23.1 ± 15.5		7.5 ± 10.8	
T3, 4	19	18.7 ± 13.4	0.39	18.7 ± 17.4	0.42
N classification					
N0	8	26.7 ± 13.6		15 ± 15.6	
N1, 2, 3	27	19.0 ± 14.3	0.21	13.6 ± 15.9	0.84
M classification					
M0	33	14.7 ± 16.1		20.1 ± 14.6	
M1	2	13.0±7.0	0.91	31.5 ± 6.5	0.31
Stage grouping					
Stage I, II	12	5.3 ± 6.6		24.9 ± 17.2	
Stage III, IV	23	17.4 ± 17.1	0.13	19.1 ± 17.2	0.38
Histology					
WHO1	3	13 ± 18.3		13.0 ± 13.4	
WHO2	19	12.3 ± 14.2		25.3 ± 13.5	
WHO3	13	18.3 ± 17.8		16.0 ± 12.1	0.99
Initial therapy					
Al ^a^	6	9.6 ± 10.9		23.5 ± 17.8	
CR ^b^	25	16.9 ± 16.7		20.6 ± 14.2	
R ^c^	4	4.0 ± 15.3		17.7 ± 12.9	0.84

^a^ Alternating cisplatin-based chemoradiotherapy. ^b^ Radiation with concurrent cisplatin-based chemotherapy. ^c^ Radiation only.

**Table 2:** Cox proportional hazard regression analysis of 35 nasopharyngeal carcinoma patients.

	Univariate Analysis		Multivariate Analysis	
Variables	Hazard Ratio (95% CI)	*p*	Hazard Ratio (95% CI)	*p*
Age (>50)	2.11 (0.46–9.65)	0.34		NI
Gender (female)	3.09 (0.81–11.78)	0.08		NI
Tumor (T3 + T4)	2.09 (0.68–6.49)	0.19		NI
Node (N2 + N3)	0.65 (0.21–2.04)	0.46		NI
Metastasis (M positive)	32.41 (2.86–367.89)	0.01	3.27 (1.57–23.67)	0.01
Stage (III + IV)	2.48 (0.55–11.2)	0.24		NI
Therapy (Al ^a^, CR ^b^, R ^c^)	0.99 (0.45–2.21)	0.99		NI
LMP1-positive	2.45 (0.82–7.31)	0.11		NI
Sema3A-positive	7.01 (1.87–26.27)	0.01	16.35 (1.42–187.94)	0.025

Factors with *p* values greater than 0.05 in the univariate models were not included (NI) in the multivariate analysis. ^a^ Alternating cisplatin-based chemoradiotherapy. ^b^ Radiation with concurrent cisplatin-based chemotherapy. ^c^ Radiation only.

## Supplementary Materials

The following are available online at https://www.mdpi.com/2076-2607/8/3/423/s1, Table S1: The expression score of Sema3A to LMP1 in each patient of NPC.
