# Supplementary material for: Overexpression of Semaphorin 3A is a Marker Associated with Poor Prognosis in Patients with Nasopharyngeal Carcinoma
Source: Microorganisms. 2020 Mar 17;8(3):423. doi: 10.3390/microorganisms8030423 (PMC7143379; doi:10.3390/microorganisms8030423)
Supplement: Supplementary file 1 [file microorganisms-08-00423-s001.pdf]

Supplementary Table S1. The expression score of Sema3A to LMP1 in each patient of NPC.

Case	Sema3A Expression Score (%)	LMP1 Expression Score (%)
1	38	6
2	18	56
3	12	18
4	22	26
5	46	0
6	22	10
7	23	0
8	1	10
9	2	0
10	23	16
11	32	41
12	45	16
13	36	13
14	25	16
15	28	37
16	23	38
17	32	39
18	5	38
19	2	0
20	12	0
21	11	0
22	32	3
23	5	0
24	16	0
25	56	10
26	3	0
27	7	0
28	34	28
29	25	20
30	41	6
31	18	0
32	2	0
33	1	3
34	5	38
35	2	0
